# Transgenic mice recapitulate the phenotypic heterogeneity of genetic prion diseases without developing prion infectivity: Role of intracellular PrP retention in neurotoxicity

**DOI:** 10.1080/19336896.2016.1139276

**Published:** 2016-02-11

**Authors:** Roberto Chiesa, Elena Restelli, Liliana Comerio, Federico Del Gallo, Luca Imeri

**Affiliations:** a Laboratory of Prion Neurobiology, Department of Neuroscience, IRCCS – “Mario Negri” Institute for Pharmacological Research, Milan, Italy;; b Department of Health Sciences, University of Milan Medical School, Milan, Italy

**Keywords:** Genetic prion disease, fatal familial insomnia, Creutzfeldt-Jakob disease, Gerstmann-Sträussler-Scheinker syndrome, PrP^C^, PrP^Sc^, transgenic mice, sleep, membrane trafficking, protein misfolding

## Abstract

Genetic prion diseases are degenerative brain disorders caused by mutations in the gene encoding the prion protein (PrP). Different PrP mutations cause different diseases, including Creutzfeldt-Jakob disease (CJD), Gerstmann-Sträussler-Scheinker (GSS) syndrome and fatal familial insomnia (FFI). The reason for this variability is not known. It has been suggested that prion strains with unique self-replicating and neurotoxic properties emerge spontaneously in individuals carrying PrP mutations, dictating the phenotypic expression of disease. We generated transgenic mice expressing the FFI mutation, and found that they developed a fatal neurological illness highly reminiscent of FFI, and different from those of similarly generated mice modeling genetic CJD and GSS. Thus transgenic mice recapitulate the phenotypic differences seen in humans. The mutant PrPs expressed in these mice are misfolded but unable to self-replicate. They accumulate in different compartments of the neuronal secretory pathway, impairing the membrane delivery of ion channels essential for neuronal function. Our results indicate that conversion of mutant PrP into an infectious isoform is not required for pathogenesis, and suggest that the phenotypic variability may be due to different effects of mutant PrP on intracellular transport.

## INTRODUCTION

**FIGURE 1.**
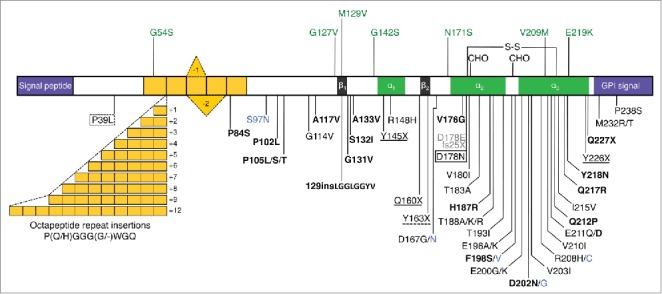
Schematic representation of the human PrP structure, with polymorphisms and reportedly pathogenic *PRNP* variants. The human (hu) PrP 1–253 structure is depicted with polymorphisms and post-translational modifications indicated above, and reportedly pathogenic mutations below. In the mature protein (huPrP 23–230) the N-terminal signal peptide (1–22) and the C-terminal signal (231–253) for attachment of the glycosyl-phosphatidyl-inositol (GPI) anchor are removed. Region 51–91 containing one nonapeptide and 4 octapeptides is in yellow; β-sheet regions (β_1_ 128–131 and β_2_ 161–164) are in black and α-helices (α_1_ 144–154, α_2_ 173–194 and α_3_ 200–228) are in green. S-S, disulphide bond between aminoacids 179 and 214; CHO, N-glycosylation sites at positions 181 and 197. All mutations are associated with a CJD phenotype except those in bold type (GSS), solid box (FFI or CJD depending on codon 129 genotype), dotted box (frontotemporal lobar degeneration-like phenotype), solid underlined (PrP-CAA), dash underlined (peripheral PrP amyloidosis with PrP-CAA), gray (sensory neuropathy) and blue (unclassified phenotype). The octapeptide repeat insertions produce variable phenotypes, including CJD, GSS- and frontotemporal dementia-like syndromes.

Genetic prion diseases are dominantly inherited neurodegenerative disorders linked to mutations in the *PRNP* gene encoding the cellular prion protein (PrP^C^), on chromosome 20. More than 60 pathogenic *PRNP* variants have been reported, including missense mutations that lead to single amino acid substitutions, expansions or deletions of a repeated sequence encoding an octapeptide motif in the N-terminal region, and stop codon mutations resulting in premature protein truncations (Fig. 1).

One striking feature of genetic prion diseases is their phenotypic heterogeneity. Different *PRNP* mutations are associated with distinct clinical and neuropathological phenotypes: genetic Creutzfeldt-Jakob disease (gCJD), fatal familial insomnia (FFI), Gerstmann-Sträussler-Scheinker (GSS) syndrome, PrP cerebral amyloid angiopathy (PrP-CAA) and PrP systemic amyloidosis.^1,2^ The disease presentation can be influenced by *PRNP* polymorphism at codon 129, where either methionine (M) or valine (V) may be present. A noteworthy example is the prion disease linked to the substitution of asparagine (N) for aspartic acid (D) at codon 178 which, depending on the aminoacid at codon 129 on the mutant allele, segregates with either FFI (D178N/M129), primarily characterized by severe sleep disorders and autonomic dysfunction, or CJD^178^ (D178N/V129), clinically identified by global cortical dementia and motor abnormalities.

How sequence variants of *PRNP* encode the information to specify distinct disease phenotypes is a central question in prion biology. The pathogenic mutations favor PrP^C^ misfolding, eventually leading to the formation of a detergent-insoluble, proteinase-K (PK)-resistant isoform (PrP^Sc^ or prion) which propagates by imprinting its abnormal conformation onto native PrP^C^ molecules.^3^ Brain tissues from different genetic prion disease patients contain protease-resistant PrP isoforms with distinct PK cleavage sites, consistent with their different conformations. When laboratory animals are inoculated intracerebrally with brain homogenates from these patients, they accumulate PrP^Sc^ molecules with the same PK resistant core as the inoculated PrP, and develop neurological diseases with different incubation times and neuropathological lesions.^4^ Thus, different mutant PrPs can give rise to conformational variants of PrP^Sc^ (prion strains) which can be propagated in laboratory animals, causing distinct brain pathologies.

However, some genetic prion diseases develop in the virtual absence of biochemically detectable PrP^Sc^, or in the presence of other abnormal forms of PrP, and are difficult to transmit or nontransmissible to laboratory animals.^2,5^ This suggests that mutant PrP has neurotoxic properties that are independent of its ability to self-replicate and that pathogenic mechanisms unrelated to prion strain propagation may govern disease manifestation in carriers of *PRNP* mutations.

To investigate the mechanisms of mutant PrP neurotoxicity, and specifically the role of the M/V 129 polymorphism in directing the disease phenotype, we developed transgenic (Tg) mouse models of CJD^178^ and FFI. We previously reported that Tg(CJD) mice, expressing the mouse PrP (moPrP) homolog of human PrP D178N/V129 (moPrP D177N/V128), closely reproduce essential features of CJD^178^.^6^ More recently, we have generated Tg(FFI) mice expressing moPrP D177N/M128, and found that they develop a fatal neurological disease different from that of Tg(CJD) mice and highly reminiscent of FFI.^7^ Comparative analysis of these models, and similarly generated Tg(PG14) mice carrying a octapeptide repeat expansion associated with GSS,^8^ indicate prion infectivity-independent mechanisms of pathogenesis and phenotypic expression of disease.

## A TRANSGENIC MOUSE MODEL OF FFI

FFI was described in 1986 by Elio Lugaresi and coworkers in a 53-year-old Italian man brought to their attention by Ignazio Roiter, a physician who had been investigating a “peculiar fatal disorder of sleep” which had run in his spouse's family since the middle of the 19th century.^9^ In addition to the large FFI kindred identified by Roiter (reported in Cortelli et al.^10^), families with the FFI mutation were later found in Italy and many countries worldwide, including France, Germany, Austria, UK, USA, Australia, Japan and China. The FFI mutation has a high penetrance^11^—more than 90% of the carriers will develop the disease, which usually manifests in the fifth decade of life. Analysis of 46 cases belonging to the Italian kindred indicated that the risk of developing FFI is highest between 50 and 55 y of age.^12^ So far only 2 carriers in this kindred reached old age (>75 years) without developing FFI (I. Roiter, personal communication).

Disruption of sleep is the most striking clinical feature of FFI.^13^ It begins with the inability to initiate and maintain sleep. As the disease progresses patients become sleep deprived, somnolent and apathetic, and experience oneiric behaviors—peculiar hallucinatory states during which they display motor gestures related to the content of a dream. Longitudinal 24 h electroencephalography (EEG) shows an early and progressive reduction of sleep spindles and K complexes (2 EEG waveforms typical of sleep without rapid eye movements or NREM sleep), severe reduction of total sleep time, sleep fragmentation and profound disruption of its cyclic organization. Deep-sleep stages and slow-wave activity (SWA, a measure of sleep drive and depth) are largely lost, and patients enter a subwakefulness state interrupted by episodes of rapid eye movement (REM) sleep which are behaviorally linked with dream enactment.^13^ The loss of sleep is associated with increased motor activity and loss of 24 h circadian motor rhythm. Patients also present progressive increases in heart and breathing rates, systemic arterial pressure, and core body temperature, indicative of sympathetic hyperactivity. Death usually occurs within 2 y from the initial clinical signs, with individuals carrying M at codon 129 on the wild-type (WT) *PRNP* allele (M/M129 homozygotes) having a shorter course than M/V129 heterozygotes.^13^ Neuropathological examination at autopsy shows predominant degeneration of the anterior ventral and mediodorsal nuclei of the thalamus, with variable involvement of the cerebral cortex and cerebellum, and moderate astrogliosis. The brains of FFI patients contain small amounts of PK-resistant PrP, which has an apparent molecular mass of 19 kDa after deglycosylation. Widespread PK-resistant PrP deposits can be detected by immunohistochemistry, especially in the cases of long duration.^14^

We produced Tg(FFI) mice using the experimental strategy previously used to generate Tg(CJD) and Tg(PG14) mice.^6,8^ The cDNA encoding moPrP D177N/M128 was cloned into the MoPrP.Xho transgenic vector (also known as half genomic PrP) which contains a 12 kb fragment of the moPrP gene (*Prnp*), including the promoter and intron 1, and drives the expression of transgenic PrP with a tissue pattern similar to that of endogenous moPrP.^6^ The transgene was injected into the pronuclei of fertilized eggs from an F_2_ cross of C57BL/6J x CBA/J F_1_ parental mice.

In a first set of experiments, we used a version of moPrP D177N/M128 in which leucine 108 and valine 111 were replaced with methionine residues. This generates the epitope for monoclonal antibody 3F4, which is normally found in hamster and human but not in mouse PrP, and which we used in our previous Tg models to distinguish transgenic from endogenous moPrP.^6,8^ However, since the 3F4 epitope makes moPrP less prone to prion-induced conversion,^15-17^ we reasoned that it could hamper spontaneous conversion of mutant PrP to infectious PrP^Sc^; therefore we also constructed Tg(FFI) mice expressing untagged PrP. The Tg founders were backcrossed for > 9 generations with an inbred colony of C57BL/6J/*Prnp*^0/0^ (PrP knockout, KO) mice, so they expressed mutant but not endogenous wild-type PrP and had a homogeneous genetic background.^7^ Parallel with the Tg(FFI) mice, we also produced new lines of Tg(CJD) mice expressing untagged moPrP D177N/V128.^7^ These mice synthesized detergent-insoluble, protease-resistant PrP molecules in their brains, and developed a neurological disease indistinguishable from that previously described in Tg(CJD) mice expressing 3F4-tagged mutant PrP.^6^

Tg(FFI) mice expressing mutant PrP at physiological levels developed mild neurological signs and lived as long as nontransgenic controls, consistent with previous observations in knockin mice in which 3F4-tagged moPrP D177/M128 was expressed from the endogenous *Prnp* locus.^17^ In contrast, Tg(FFI) mice expressing mutant PrP at twice the endogenous level or more developed a progressive and invariably fatal neurological disease, and accumulated detergent-insoluble, protease-resistant PrP in their brains, yielding a 19 kDa C-terminal fragment after mild PK digestion and deglycosylation, like in FFI patients.^7^

**FIGURE 2.**
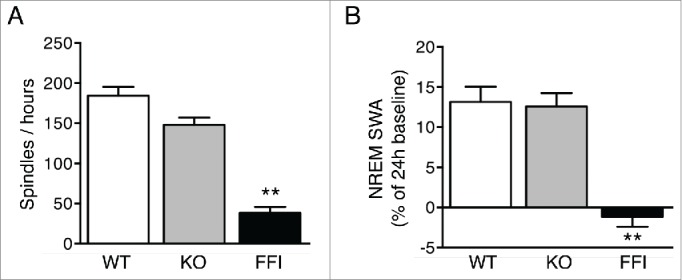
Sleep spindles and slow-wave activity are reduced in Tg(FFI) mice. Number of sleep spindles per hour (A) and normalized SWA during NREM sleep (B) were measured as described.^7^ Data are the mean ± SEM of 8 non-Tg/*Prnp*^+/+^ (WT), 10 non-Tg/*Prnp*^0/0^ (KO) and 9 Tg(FFI)/*Prnp*^0/0^ (FFI). **p < 0.01 by one-way ANOVA with Bonferroni's correction.

**FIGURE 3.**
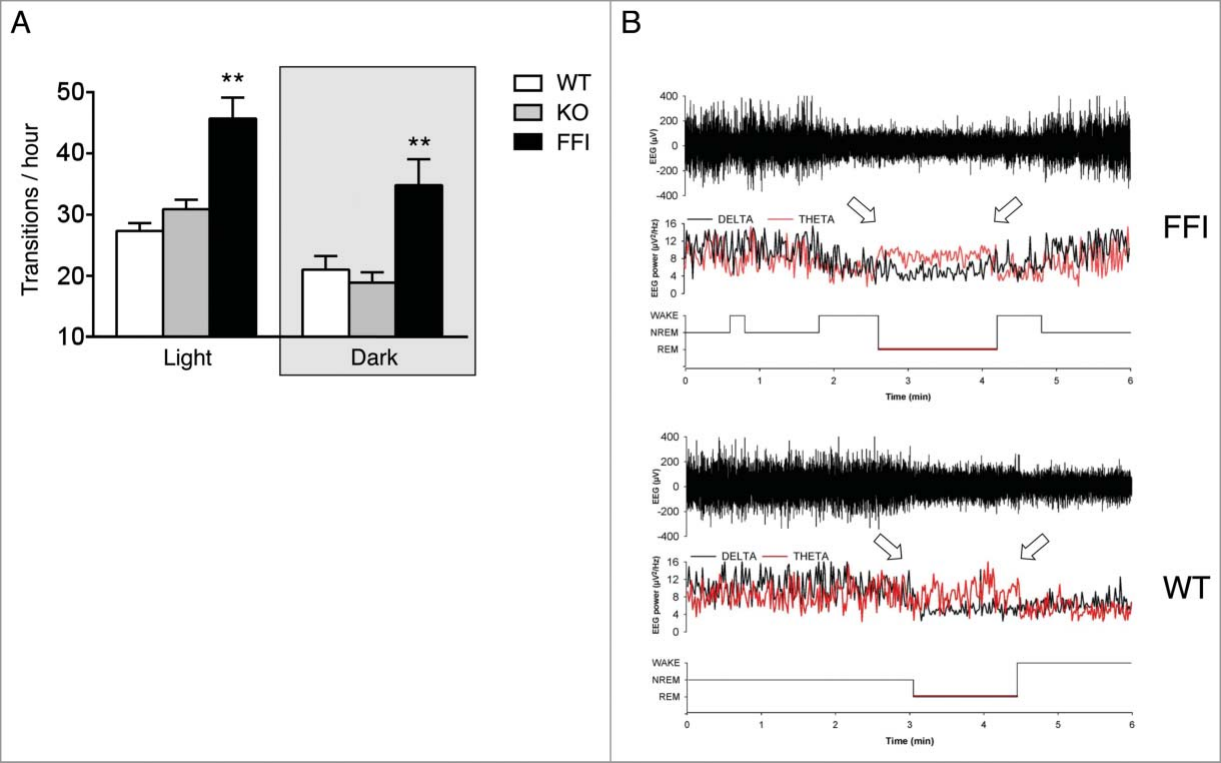
Sleep continuity and organization are affected in Tg(FFI) mice. (A) The number of transitions between wake, REM and NREM sleep, during the phases of the light-dark cycle were measured as described.^7^ Data are the mean ± SEM of 8 non-Tg/*Prnp*^+/+^ (WT), 10 non-Tg/*Prnp*^0/0^ (KO) and 9 Tg(FFI)/*Prnp*^0/0^ (FFI). **p < 0.01 by mixed model for repeated measures followed by between strains one-way ANOVA with Bonferroni's correction. (B) Whereas a Tg(FFI)/*Prnp*^0/0^ (FFI) mouse enters REM sleep directly from wakefulness (left arrow), as shown by the hypnogram (lower trace), in a non-Tg/*Prnp*^+/+^ (WT) mouse REM sleep is preceded by an episode of NREM sleep. Top to bottom: EEG (electroencephalogram), EEG power in the delta (0.5 – 4 Hz, black line) and theta (6 – 9 Hz, red line) bands, and the related hypnogram. Arrows indicate the beginning and end of a REM sleep phase. Panel B is from ref. 7, Creative Commons Attribution (CC BY) license.

Tg(FFI) mice had profound sleep alterations reminiscent of those seen in human FFI.^7^ Longitudinal 24h monitoring and spectral EEG analysis showed a marked reduction of sleep spindle density compared to nontransgenic mice (Fig. 2A). SWA during NREM sleep was significantly reduced in Tg(FFI) mice (Fig. 2B). The mutant mice also presented a profound disruption of sleep continuity and organization. They had a larger number of transitions between the different behavioral states (wake, REM and NREM sleep) than controls (Fig. 3A), indicating broken, non-restorative sleep. In addition, in Tg(FFI) mice approximately a quarter of REM sleep episodes started directly from wakefulness, instead of being preceded by NREM sleep as normally occurs (Fig. 3B). This was reminiscent of the sudden-onset episodes of REM sleep that intrude into wakefulness in FFI patients.

**FIGURE 4.**
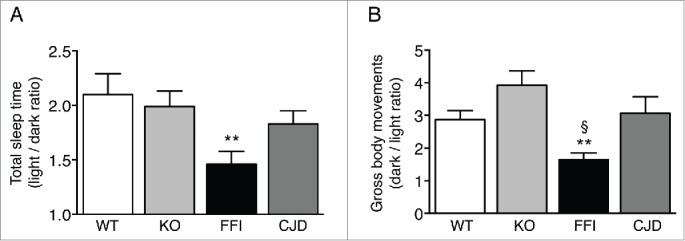
Circadian organization of sleep and motor activity is lost in Tg(FFI) but not Tg(CJD) mice. (A) Ratio of the total amounts of time animals spent asleep (NREM + REM) during the phases of the light-dark cycle. (B) Ratio of the gross body movements made by the animals during the phases of the light-dark cycle. Data were from 8 non-Tg/*Prnp*^+/+^ (WT), 10 non-Tg/*Prnp*^0/0^ (KO), 9 Tg(FFI)/*Prnp*^0/0^ (FFI), and 9 Tg(CJD)/*Prnp*^0/0^ (CJD), as described.^7^ Data are the mean ± SEM. **p < 0.001 vs. WT and KO in A, and vs. KO in B; ^§^p < 0.05 vs. WT in B; by one-way ANOVA, followed by post-hoc Fisher's least significant difference (LSD) test.

Like in FFI patients, the circadian organization of sleep and motor activity was lost in Tg(FFI) mice. Mice are nocturnal animals and sleep about twice as much during the day as during the night. Tg(FFI) mice slept only 50 percent more than controls (Fig. 4A). Moreover, compared to nontransgenic mice which moved 3–4 times more during the night than during the day, Tg(FFI) mice moved only about 1.5 times more (Fig. 4B).

Sleep-wake disturbances different from those seen in FFI have been reported in sporadic CJD and in CJD^178^ and are faithfully modeled by Tg(CJD) mice.^6,18^ The alterations in sleep and circadian rhythm in Tg(FFI) mice differed significantly from those in Tg(CJD) mice. Whereas in Tg(FFI) mice the circadian organization of sleep and motor activity was lost, sleep was fragmented, and theta activity (the EEG hallmark of rodent REM sleep) was reduced,^7^ in Tg(CJD) mice the circadian organization of sleep and motor activity was preserved (Fig. 4), and sleep continuity and its EEG patterns were not altered.^6,7^ Thus Tg(FFI) and Tg(CJD) mice recapitulated the specific sleep alterations of the corresponding human diseases.

Other clinical symptoms of FFI include dysautonomia, disturbances of attention and vigilance, difficulties with the temporal ordering of events and spatial disorientation, gait abnormalities, ataxia and other motor deficits. Autonomic cardiac control was altered in Tg(FFI) mice.^19^ They were also impaired in recognition and spatial working memory, and showed sensorimotor deficits, abnormal gait and ataxia.^7^ Neuropathological analyses indicated moderate gliosis and PK-resistant PrP deposits in the thalamus and other brain regions. MRI detected thalamic and cerebellar atrophy.^7^ Thus, many salient aspects of human FFI were recapitulated in transgenic mice.

## SPONTANEOUS PRION GENERATION IS NOT REQUIRED FOR MANIFESTATION OF DISEASE IN TRANSGENIC MICE

To answer the question whether prion infectivity was generated *de novo* in the brains of Tg(FFI) and Tg(CJD) mice, 10% brain homogenates from clinically ill mice were inoculated intracerebrally into C57BL/6J mice and Tg*a*20 mice which overexpress moPrP WT and are highly sensitive to prions. Since homology between PrP^Sc^ in the inoculum and PrP^C^ in the recipient mice may increase the chance of disease transmission,^20,21^ the brain homogenates from Tg(FFI) and Tg(CJD) mice expressing 3F4-tagged mutant PrP were also inoculated into Tg(WT-E1) mice overexpressing 3F4-tagged moPrP WT, and into Tg mice, which express low levels of 3F4-tagged moPrP D177N and do not spontaneously become ill.^6,8^ None of the inoculated animals developed signs of neurological disease and either died of intercurrent illnesses or were euthanized near the end of their normal lifespan. Biochemical analysis found no PrP^Sc^ in their brains.^7^

To test the possibility that Tg(FFI) and Tg(CJD) brains contained prions below the threshold of detection of our bioassay, the brain homogenates were subjected to serial protein misfolding cyclic amplification (PMCA), which allows highly efficient prion replication *in vitro* and can amplify the equivalent of a single molecule of PrP^Sc^. No PrP^Sc^ was detectable in the PMCA reactions by either biochemical analysis or bioassay in Tg*a*20 mice.^7^ Thus the manifestation of disease in Tg(FFI) and Tg(CJD) mice was not associated with spontaneous prion formation. This was also the case for Tg(PG14) mice,^16^ and other models of GSS^22,23^ (J. A. Mastrianni, personal communication), indicating that neurotoxic mechanisms are operative in Tg mice that produce different diseases independently of prion generation and spread.

## POTENTIAL ROLE OF INTRACELLULAR PRP ACCUMULATION IN PHENOTYPIC EXPRESSION OF DISEASE

The lack of prion infectivity in Tg(FFI), Tg(CJD) and Tg(PG14) mice indicates that conversion of the mutant PrPs into distinct self-replicating PrP^Sc^ isoforms is not the cause of phenotypic variation. What is it then that produces different diseases in these models? Disease-specific features are seen in independently generated lines of Tg(FFI), Tg(CJD) and Tg(PG14) mice,^6-8^ strongly indicating that they are encoded by the mutant PrPs, rather than non-specific effects of random transgenesis. Moreover, backcrossing the different mutant mice to the same inbred strain of C57BL/6J/*Prnp*^0/0^ mice ruled out non-specific effects of mixed genetic backgrounds or genetic drift.

Investigation of PrP metabolism and localization in primary neurons and transfected cells showed that the PG14 and D177N mutants are delayed in their biosynthetic maturation in the endoplasmic reticulum (ER), and accumulate abnormally in the secretory pathway,^24,25^ but do not activate ER stress-related maladaptive responses.^26,27^ PG14 and D177N/V128 PrPs were mainly found in the ER, while D177N/M128 localized preferentially to the Golgi.^6,7^ This may reflect differences in how these mutants acquire abnormal conformations and aggregate during secretory transport. There is evidence, in fact, that PG14, D177N/V128 and D177N/M128 PrPs differ in their monomeric structures and states of aggregation,^28^ and aggregation and intracellular retention are closely related.^29^

In Tg(PG14) and Tg(CJD) mice, ER retention of mutant PrP impairs the synaptic delivery of voltage-gated calcium channels (VGCCs) through a physical interaction with the VGCC α_2_-δ subunit; this leads to reduced expression of functional channels on the plasma membrane, and defective cerebellar neurotransmission and motor control.^30^ We therefore hypothesized that intracellular retention of mutant PrP might affect the trafficking and function of other PrP^C^-interacting ion channels, and that mutants that tend to accumulate in different intracellular compartments might affect trafficking in different ways, producing specific neurotoxic effects.^27,31^

PrP^C^ interacts physically with certain subunits of N-methyl-D-aspartate (NMDA) and α-amino-3-hydroxy-5-methyl-4-isoxazolepropionic acid (AMPA) receptors, and these interactions are important for normal neuronal physiology and survival.^32,33^ The assembly and trafficking of these receptors are finely tuned in the ER and Golgi. Our preliminary observations indicate that PG14, D177N/V128 and D177N/M128 alter NMDA and AMPA receptor trafficking in different ways. They also interact differently with receptor subunit isoforms expressed in functionally distinct neurons of the brain^33^ (and our unpublished results). Thus PG14, D177N/V128 and D177N/M128 may have different effects on the function and survival of different neurons—hence on the clinical presentation of disease— depending on where in the secretory pathway they preferentially accumulate, and how this interferes with the transport of the molecules they interact with.

## CONCLUDING REMARKS

A number of mouse models of genetic prion disease have been generated, most of which recapitulate specific features of the human disorders without developing *bona fide* prions,^6-8,16,17,21-23,34-36^ perhaps underscoring the difficulty of reconstituting in short-lived animals a molecular transformation that in humans may take decades or not even occur. However, the lack of infectivity does not invalidate these models. In fact, it is an incidental advantage, since it permits analysis of the primary pathogenic consequences of mutant PrP misfolding independently of toxic effects downstream of PrP^Sc^ replication.

Our findings indicate a key pathogenic role of PrP misfolding in the secretory pathway. By accumulating intracellularly, the mutant PrPs affect the trafficking of PrP^C^-interacting ion channels, causing alterations in neurotransmission and behavioral abnormalities.^30^ Our preliminary evidence indicates that different mutants cause specific alterations of secretory transport, suggesting a neurotoxic modality that may explain the phenotypic heterogeneity of genetic prion diseases.^27^ It is now important to identify the molecules whose transport is disrupted by the different mutants, to clarify how this leads to selective neurotoxicity in animal models, and to test whether the same mechanisms are operative in humans. These important challenges will not only cast light on the physiopathology of genetic prion diseases but also help point the way to devising targeted therapeutic approaches for these devastating disorders.
